# Treatment of Rheumatoid Arthritis-Associated Interstitial Lung Disease: Lights and Shadows

**DOI:** 10.3390/jcm9041082

**Published:** 2020-04-10

**Authors:** Giulia Cassone, Andreina Manfredi, Caterina Vacchi, Fabrizio Luppi, Francesca Coppi, Carlo Salvarani, Marco Sebastiani

**Affiliations:** 1 Clinical and Experimental Medicine PhD Program, University of Modena and Reggio Emilia, 41124 Modena, Italy; giulia.cassone@unimore.it; 2 Rheumatology Unit, IRCCS Arcispedale Santa Maria Nuova, Azienda Unità Sanitaria Locale-IRCCS di Reggio Emilia, 42100 Reggio Emilia, Italy; 3 Chair and Rheumatology Unit, University of Modena and Reggio Emilia, Azienda Ospedaliero-Universitaria Policlinico di Modena, 41124 Modena, Italy; andreina.manfredi@gmail.com (A.M.); carlo.salvarani@ausl.re.it (C.S.); 4 Clinical and Experimental Medicine PhD Program, University of Modena and Reggio Emilia, 41124 Modena, Italy; caterina.vacchi@unimore.it; 5 Respiratory Unit, University of Milano Bicocca, S. Gerardo Hospital, 20900 Monza, Italy; fabrizio.luppi@unimib.it; 6 Department of Cardiology, University of Modena and Reggio Emilia, Azienda Ospedaliero-Univesitaria Policlinico di Modena, 41124 Modena, Italy; coppi.francesca@aou.mo.it

**Keywords:** rheumatoid arthritis, interstitial lung disease, therapy, DMARDs, antifibrotic agents

## Abstract

Rheumatoid arthritis (RA) is a chronic and systemic inflammatory disease affecting 0.5–1% of the population worldwide. Interstitial lung disease (ILD) is a serious pulmonary complication of RA and it is responsible for 10–20% of mortality, with a mean survival of 5–8 years. However, nowadays there are no therapeutic recommendations for the treatment of RA-ILD. Therapeutic options for RA-ILD are complicated by the possible pulmonary toxicity of many disease modifying anti-rheumatic drugs (DMARDs) and by their unclear efficacy on pulmonary disease. Therefore, joint and lung involvement should be evaluated independently of each other for treatment purposes. On the other hand, some similarities between RA-ILD and idiopathic pulmonary fibrosis and the results of the recent INBIULD trial suggest a possible future role for antifibrotic agents. From this perspective, we review the current literature describing the pulmonary effects of drugs (immunosuppressants, conventional, biological and target synthetic DMARDs and antifibrotic agents) in patients with RA and ILD. In addition, we suggest a framework for the management of RA-ILD patients and outline a research agenda to fill the gaps in knowledge about this challenging patient cohort.

## 1. Introduction

Rheumatoid arthritis (RA) is a chronic inflammatory disease affecting 0.5–1% of the population worldwide [1]. It is characterized by symmetrical erosive synovitis and progressive disability, often complicated by extra-articular manifestations. Among them, lung involvement is common, and it can include a wide spectrum of disorders, ranging from airways and pleural disease, bronchiectasis, and nodules, to infection and drug toxicity (Table 1) [2,3,4].

Interstitial lung disease (ILD) is a serious pulmonary complication in RA, with a negative impact on overall prognosis and utilization of healthcare resources [5,6,7,8]. About 10% of the RA patients develop a clinically significant ILD that is responsible for 10–20% of mortality, with a mean survival of 5–8 years [5,6,7,8]. However, the real incidence of RA-related ILD (RA-ILD) is still unknown, and a variable prevalence has been reported in the literature [5,6,7,8]. The usual interstitial pneumonia (UIP) is the predominant histological/radiological pattern of RA-ILD, reported in up to 66% of cases [9,10,11,12,13,14,15,16,17,18,19] (Table 2). ILD can occur at any point in the natural history of RA, sometimes before the appearance of joint involvement [14,20,21]. Since its clinical manifestations usually appear in the advanced stages of lung disease, early diagnosis of RA-ILD is challenging.

Moreover, in patients with non-symptomatic RA-ILD, a clinical, functional and radiologic follow-up of the lung is mandatory to identify patients with progressive disease.

In fact, the progression and the severity of lung involvement are the two main factors to consider when decision-making on treatment is needed. Histopathologic/radiologic pattern also plays a pivotal role. High-resolution computed tomography (HRCT) showed a high correlation with the histologic pattern, especially in UIP subtypes, therefore, it is now considered the gold-standard for the diagnosis and a lung biopsy is performed in doubtful cases only [22]. However, HRCT can sometimes fail to correctly identify the pulmonary pattern in clinical practice, since it lacks to show both some pathologic findings such as lymphoid hyperplasia and peribronchiolar lesions and overlap UIP and non-UIP patterns [23,24,25].

The decision to start treatment is also influenced by a patient′s age, gender, the worsening of symptoms or pulmonary function tests (PFTs), and the presence of comorbidities that might increase the risk of adverse events (diabetes mellitus, osteoporosis, etc.) [20,21,26,27,28,29,30,31,32]. Moreover, recent studies showed that some radiological HRCT findings such as honeycombing, correlate with a poor prognosis, but the role of the ILD pattern in the therapeutic choice is still uncertain [23,33].

The therapeutic approach to RA-ILD patients is further complicated by some unresolved matters. First, both conventional and biologic disease-modifying anti-rheumatic drugs (DMARDs) have been implicated in the development of drug-related pulmonary toxicity with conflicting data (Table 1; drug toxicity) [21,28,33,34,35,36]; secondly, there is no evidence that the treatment of RA could be effective also on lung involvement. On the contrary, immunosuppressive drugs employed in connective tissue diseases (CTD) or antifibrotic drugs approved for idiopathic pulmonary fibrosis (IPF) are not effective on arthritis.

Summarizing, to treat ILD secondary to RA is not the same as treating RA in patients with concomitant ILD.

In the absence of controlled studies, the therapeutic approach to RA-ILD is still debated and based on empirical approaches dependent on retrospective studies and case series. Several therapeutic agents have been suggested, but nowadays there are no therapeutic recommendations for the treatment of RA-ILD.

The latest American College of Rheumatology (ACR) and European League Against Rheumatism (EULAR) guidelines for the management of RA do not specifically consider patients with RA-ILD, suggesting the need for a multidisciplinary approach [37,38], while the National Institute for Health and Care Excellence (NICE) and Spanish Society of Rheumatology have proposed national recommendations, suggesting the use of abatacept and rituximab in patients with RA-ILD, and advising against the use of TNF inhibitors [39,40].

Due to the absence of evidence for RA-ILD treatment and its potential adverse effects, the decision to treat should be based on the balance between its benefits and the burden of the disease in every single patient. In asymptomatic patients with non-progressive ILD, a “wait and see” approach is usually recommended [26,27,28].

From this perspective, we review the current literature on the treatment of patients with RA-associated ILD and discuss the unsolved problems regarding this challenging patient cohort.

To achieve our purpose, we reviewed the published studies describing the pulmonary effects of drugs (immunosuppressants, conventional, biological and target synthetic DMARDs and antifibrotic agents) in patients with RA and ILD, focusing on the articles where lung involvement was specifically investigated.

Additionally, we suggest a framework for the management of RA-ILD and outline a research agenda to fill the gaps in our knowledge in this field.

## 2. Immunosuppressants

The Panther study demonstrated an increase in mortality and infections in patients with IPF treated with immunosuppressive drugs [41]. The role of immunosuppressants in usual interstitial pneumonia (UIP) in RA or CTD has not been clarified, but a better response in ILD patterns different to UIP has been suggested by some retrospective studies [42,43,44].

Therefore, RA-ILD with nonspecific interstitial pneumonia (NSIP) or organizing pneumonia (OP) patterns could have a more favorable response to immunosuppressive therapy than the UIP pattern [42,43,44].

However, some patients have unclassifiable or mixed patterns in HRCT, and a lung biopsy can also identify various histologic findings in different specimens. Unluckily, no data clarify the treatment’s response and the progression of the disease over time in these patients [45].

### 2.1. Corticosteroids

The effect of corticosteroids on patients with UIP pattern remains unclear. In the retrospective study by Song et al., 50% of 84 patients with UIP pattern treated with corticosteroids and immunosuppressants presented improvement or stabilization of lung function, but without significant differences with the untreated group [46]. Moreover, among 10 RA-ILD patients with UIP pattern, Lee et al. described the improvement of lung function in two patients and stability in three of them during a median follow-up of 4.2 years [47].

In a recent retrospective case series including 11 RA patients, therapy with pulse intravenous methylprednisolone followed by oral prednisone and tacrolimus appeared to be effective and well-tolerated [48].

On the other hand, corticosteroids increased the risk of serious infections in patients with RA-ILD. Zamora-Legoff found that a mean daily dose of prednisone higher than 10 mg was associated with a higher rate of infections, despite the combination with DMARDs [49].

In our cohort of RA patients, respiratory infections were associated with ILD, steroids, and the use of biologic DMARDs (bDMARDs). Among 33 patients with ILD, combination-therapy with b-DMARDs, methotrexate, and corticosteroids was significantly more frequently recorded in patients with infections [50].

### 2.2. Cyclophosphamide and Mycophenolate Mofetil

Cyclophosphamide (CYC) demonstrated a modest advantage in systemic sclerosis-related ILD (SSc-ILD) and other CTD-ILD [50,51,52,53,54], especially in combination with methylprednisolone pulses, although a metanalysis doubted the efficacy of CYC in SSc-related lung fibrosis [55].

There are no controlled clinical trials for CYC in RA-ILD, but it is used in clinical practice despite its limited efficacy data [46,56,57,58,59], especially in the case of rapidly progressive ILD. Recently, a Chinese retrospective study analyzed the factors associated with progression and survival in 266 RA-ILD patients, observing a better survival in the group treated with CYC [60].

Since CYC shows little benefit on RA joint involvement, it is usually associated with corticosteroids or other immunosuppressants [46,56,57,58,59,60].

Mycophenolate mofetil (MMF) is considered the main alternative to CYC as a first-line agent or a possible maintenance therapy in CTD-ILD, with a lower rate of side effects than CYC [56,61,62,63].

No studies directly compare these two drugs in RA-ILD patients, as well as no data being available to recommend MMF in RA-ILD. Saketkoo et al. described a clinical improvement and stability of functional and radiological lung assessment in a small case series of 3 RA-ILD patients [63]. In another retrospective study, MMF induced the stability of forced vital capacity (FVC) and the diffusing capacity of the lungs for carbon monoxide (DLCO) [62].

In 2016, a retrospective study from the UK found better survival in patients treated with MMF than azathioprine (AZA). The relative risk of death for any cause was increased for patients on prednisone, unaltered for AZA, and decreased for MMF. The Authors suggested a better outcome with MMF rather than CS or AZA for the treatment of RA-ILD [64]. Unlikely, both CYC and MMF are ineffective for the articular manifestations of the disease.

## 3. Conventional Disease-Modifying Antirheumatic Drugs (cDMARDs)

Disease-modifying antirheumatic drugs, both conventional (cDMARDs) and biologic (bDMARDs), have been demonstrated to improve the joint involvement of RA, but their impact on extra-articular manifestations of the disease, mainly ILD, is unclear. Case reports, case series, and data from registries or retrospective studies demonstrated a wide spectrum of pulmonary effects (Table 1; drug toxicity), including improvement, but also the development and worsening of ILD [34,46,65,66].

Only a few reports describe the use of cDMARDs as a treatment for ILD in patients with RA (Table 3).

### 3.1. Methotrexate

Methotrexate (MTX) has been associated with acute hypersensitivity pneumonia and with chronic ILD. However, recent studies and meta-analyses suggested that MTX-hypersensitivity pneumonia is less common than previously thought, and interestingly, no episodes of MTX related hypersensitivity pneumonia have been recorded in controlled trials after 2001 [67]. Moreover, the association between MTX and ILD development has been recently questioned [5,67,68,69,70].

Kiely described data by ERAN and ERAS registries, showing no increased risk of ILD in RA patients treated with MTX; even better, exposure to MTX was associated with a significantly reduced risk of incident RA-ILD [70].

In 2014, a meta-analysis including 8584 participants from 22 double-blind, randomized, controlled trials demonstrated an increased risk of total adverse respiratory events relative to comparator agents. In particular, MTX was associated with an increased risk of total infectious adverse respiratory events, but not with noninfectious respiratory adverse events, including ILD and MTX-related pneumonitis [71]. Finally, Rojas-Serrano has recently observed a longer survival in RA-ILD patients treated with MTX compared to other cDMARDs [72].

One paper by the same Authors describes the use of MTX and leflunomide (LEF) for the treatment of ILD in 40 RA patients. Patients received prednisone 1 mg/kg/day associated with MTX in 18 patients and LEF or AZA, or both, in 22. After a 4-month follow-up, FVC improved in both groups [65].

Although recent studies questioned the role of MTX as a causative agent in lung involvement [67,68,69,70,71,72], it may be appropriate to conduct tight monitoring of lung function in patients with an established diagnosis of ILD in treatment with MTX [72,73]. However, MTX remains one of the central drugs in the treat-to-target approach to RA, so treatment should not be delayed or limited in active and progressive RA [37].

### 3.2. Leflunomide

Leflunomide has been associated with rapid onset hypersensitivity pneumonia and new-onset or progression of pre-existing ILD, with discordant published data.

In a Japanese cohort, 1.2% of 5054 RA patients who received LEF had a new-onset and/or exacerbation of ILD. Pre-existing ILD was the most important risk factor for LEF-induced ILD [74]. Similar results were achieved by Ju et al. in 1010 Korean patients, but no deaths due to ILD were detected [75].

In 2006, Suissa reported an increased risk of worsening or new onset of ILD with LEF, but only in patients with previous use of MTX or preexisting ILD [76].

Two systematic reviews found conflicting results. Conway et al. showed no association between LEF and increased risk of total or infectious respiratory adverse events in eight controlled trials [77]; on the contrary, a previous systematic review demonstrated an association between the appearance or worsening of ILD and LEF. Bilateral ground-glass opacities and diffuse alveolar damage were the most common radiologic and histopathologic findings of ILD secondary to leflunomide [78].

### 3.3. Azathioprine

In 1977, a case report described a patient with RA biopsy-proven ILD that improved after azathioprine (AZA) administration [79]. More recently, two retrospective studies described RA-ILD-UIP patients treated with corticosteroids and cDMARDs, including AZA, without conclusive results [46,65].

On the other hand, pulmonary toxicity has been also described for AZA [80]. In 2016, Oldham et al. studied adverse events related to AZA in patients with fibrotic CTD-ILD, including 15 patients with RA-ILD. Finally, AZA was compared with MMF, demonstrating a marginally better efficacy but a higher rate of side effects [81].

### 3.4. Sulfasalazine, Hydroxychloroquine, and Penicillamine

Lung toxicity is a rare side effect of sulphasalazine. In the past decades, numerous case reports have been published implicating sulphasalazine in acute lung toxicity, namely interstitial pneumonitis and eosinophilic pneumonia. Many patients with suspected sulphasalazine-induced lung disease improved within a few weeks after drug withdrawal [82]. No data are available regarding the pulmonary toxicity of hydroxychloroquine. Although D-penicillamine could induce acute hypersensitivity pneumonitis [83,84], its use in the treatment of RA-ILD has been also anecdotally described [85].

### 3.5. Calcineurin Inhibitors (Cyclosporin, Tacrolimus)

Ciclosporin has been used in the treatment of RA-ILD with slight efficacy [46,86,87,88,89]. Some case reports described improvement or stability of ILD in a total of four RA patients treated with cyclosporin [80,86,87,88].

Tokano et al. describe a case series of 25 patients with various rheumatic diseases and steroid-resistant ILD, treated with cyclosporin A. Of the four patients with a diagnosis of RA, only one showed a transient response, while two patients died and the latter showed no response [89].

Tacrolimus has been successfully used in patients with ILD related to inflammatory myositis, such as dermatomyositis and anti-synthetase syndrome, also when presenting as acute respiratory distress syndrome [90,91,92].

In 2018, Yamano et al. treated 26 patients with ILD, including 11 with RA, with tacrolimus and steroids. After a 12-month follow-up, PFTs and dyspnea significantly improved, without remarkable life-threatening adverse events [48].

However, the use of calcineurin inhibitor is often limited by their side effects and their efficacy in RA-ILD remains undefined and needs more dedicated studies (Table 3).

## 4. Biological Disease-Modifying Antirheumatic Drugs (bDAMRDs)

Almost all of the bDMARDs have been associated with lung toxicity; however, their ability to improve lung function and stabilize pulmonary symptoms have been also described [34,35,36].

The possible effectiveness of bDMARDs in RA-ILD has been described in only a few retrospective studies and as anecdotical reports. Most of the available data in this field in current literature describe the pulmonary effect of bDMARDs used for RA in patients with concomitant ILD.

### 4.1. Tumour Necrosis Factor Alpha Inhibitors

Tumour necrosis factor-alpha inhibitor (TNFi) may have both profibrotic and antifibrotic effects, the imbalance between these two roles might trigger fibrosis or stabilize ILD [93,94,95].

In fact, transgenic mice over-expressing TNF-alpha develop interstitial pneumonitis resembling IPF. TNF-alpha upregulates the expression of transforming growth factor-beta 1 in vitro and in an animal model, resulting in chronic inflammation and lung fibrosis [93]; but, on the contrary, the TNF-alpha supplementation ameliorates the lung function and architecture in TNF-alpha (−/−) mice with bleomycin-induced lung fibrosis [95].

TNFi has been associated by many Authors to new onset or exacerbation of RA-ILD [96,97,98,99,100,101,102,103,104], and the British Society of Rheumatology specifically cautioned prescribing TNFi to patients with RA-ILD [105]. Recently, the NICE and Spanish Society of Rheumatology contraindicated TNFi in this population [39,40]

Despite these observations, other studies confuted lung toxicity for TNFi and showed that these drugs can stabilize or even improve pulmonary interstitial disease [34,106,107]. For example, an American cohort study observed that, compared with cDMARDs, the use of TNFi did not increase the occurrence of ILD among RA patients [106].

A British national prospective observational study of 367 patients with pre-existing RA-ILD found that the mortality in patients with RA-ILD was not increased by treatment with TNFi compared with cDMARDs. However, the proportion of deaths attributable to RA-ILD was higher in patients treated with TNFi therapy (34%) [96].

Finally, in the only prospective study available, Detorakis evaluated, in RA patients with or without ILD, the effects of TNFi on lung parameters, observing that the ILD extent score remained unchanged both in the TNFi and cDMARDs groups. There was no exacerbation of ILD, nor new ILD onset in patients without pre-existing ILD. Moreover, TNFi induced an improvement of small airways disease [108].

On the contrary, Nakashita described a potentially negative role of TNFi in RA-ILD patients. He described 14 interstitial disease events on 46 RA-ILD patients treated with TNFi; among them, four patients developed generalized lung disease and two died from ILD progression [109]. The same Authors did not observe an increase in the prevalence of ILD progression in patients treated with tocilizumab and abatacept, whereas prevalence of 3% of new ILD appearance and 24% of ILD worsening were described in TNFi users [109].

Curtis did not find significant differences in the risk of ILD and its related complications among RA patients treated with a second-line biologic therapy after TNFi, comparing second-line TNFi agents or bDMARDs with other mechanisms of action [110].

#### 4.1.1. Infliximab

Only a few case reports describe the positive effect of infliximab on RA-ILD. Vassallo described an improvement of dyspnea and stabilization of PFTs in a patient with RA-ILD refractory to corticosteroid, after 12 months of infliximab treatment [107]. Bargagli reported another similar case in 2004 [111], while Antoniou et al. identified a good response to infliximab in a case series of three previously progressive RA-ILD patients [112].

Otherwise, there are many reports about the possible iatrogenic role of infliximab in the development or exacerbation of RA related ILD [97,101]. In a post-marketing surveillance study in Japanese RA patients, interstitial pneumonitis was observed in 25 patients (0.5%), after a mean of 2.8 infusions of infliximab [103] and respiratory disorders were the most common serious adverse drug reactions.

#### 4.1.2. Adalimumab

As for the other TNFi, data was derived by post-marketing surveillance reports. The Japan College of Rheumatology, on 3000 RA patients treated with adalimumab, described the occurrence of interstitial pneumonia in 0.6% of patients [98]. In another retrospective Japanese study on 200 RA patients treated with adalimumab, respiratory disorders were the third most common adverse event, represented by interstitial lung lesions in three patients and organizing pneumonia in two [113].

A case report in 2011, described conflicting actions of adalimumab in the same patient with RA-ILD, suggesting that the drug might be effective against RA-ILD, but may also have caused drug-induced ILD [114]. Case reports also describe an anecdotal association between ILD and the use of adalimumab [115,116].

#### 4.1.3. Etanercept

Etanercept has been evaluated in a randomized controlled trial in the treatment of IPF, but without obtaining significant differences from placebo in disease progression nor the change of predicted FVC from baseline [117].

Only two case reports described possible effectiveness of etanercept in females with RA-ILD [118,119]. In the first, treatment with etanercept improved symptoms and physical capacities in a girl with juvenile chronic arthritis and pulmonary interstitial disease [118]. More recently, Wang et al. described a sustained improvement in symptoms, PFTs, and high-resolution computer tomography (HRCT) findings after etanercept treatment in a 52-year-old woman with RA-ILD refractory to corticosteroids and azathioprine [119].

In 2012, a short review described 12 RA-ILD patients treated with etanercept, six with pre-existing ILD and six with newly-diagnosed ILD. Among them, eight patients developed a severe ILD (without concomitant use of MTX) and two patients died [120].

Moreover, exacerbation of pre-existing ILD during etanercept therapy in RA patients was described [121,122,123]. In a real-life surveillance report published in 2011, among 13,894 patients, the most frequent serious adverse events were pneumonia (0.8%) and interstitial lung disease (*n* = 77, 0.6%) [124]. Two years later, the same Authors observed that etanercept in association with MTX showed significantly lower incidence rates for total adverse events, including ILD, than etanercept alone or associated with DMARDs different to MTX [125].

#### 4.1.4. Golimumab and Certolizumab

**Figure 1:**
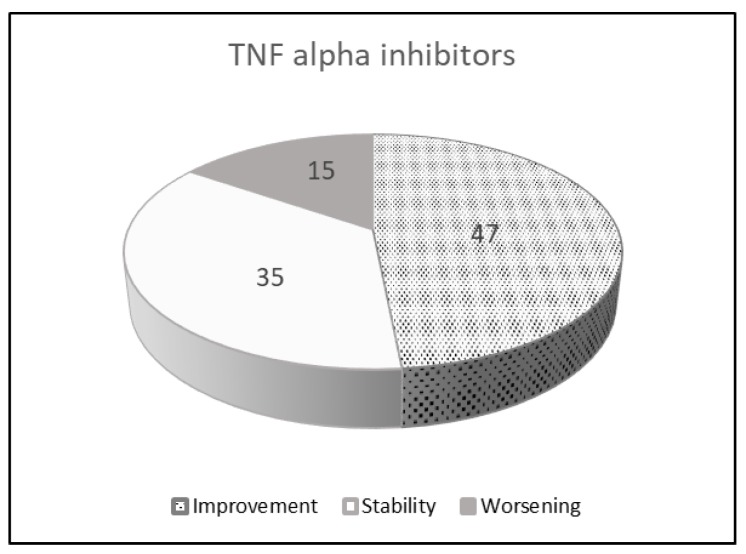
Pulmonary effects of TNFi in RA-ILD patients: a review of the literature.

No reports are describing the use of certolizumab or golimumab in the treatment of RA-ILD patients, but, on the contrary, a possible relationship between these drugs and new-onset or acute exacerbation (AE) of ILD has been described [100,126,127,128,129]. In 2017, a post-marketing surveillance study from 2579 Japanese patients treated with certolizumab reported an event rate of ILD of 1.22 per 100 patient-year (Table 4 and Figure 1) [130].

### 4.2. Abatacept

Increasing interest in abatacept in RA-ILD is emerging in the last years. In a murine model, abatacept (ABA) significantly reduced fibrogenic marker levels, T-cell proliferation, and M1/M2 macrophage infiltration in the lungs of Fra-2 mouse model, characterized by ILD and pulmonary vascular remodeling leading to pulmonary hypertension [132]. Moreover, ABA improved ILD, significantly reducing the lung density on chest HRCT and fibrosis histological score [133].

In 2014, Mera-Valera et al. used ABA in a case series of four RA-ILD patients, observing no adverse events nor deterioration of respiratory function tests [134]. However, in a previous case report, ILD worsened two days after the administration of ABA and subsequently improved after drug discontinuation in a 55-year-old man enrolled in a trial of phase III of ABA in Japan. Interstitial shadows worsened on HRCT scans taken on day 13, and the patient withdrew from the trial. [135].

Ye used corticosteroids and ABA to control both joint and pulmonary disease in a RA patient who developed ILD during treatment with rituximab and MTX. The PFTs parameters improved despite a reduction of the steroid dose to 5 mg daily. [136].

Nowadays, there are six retrospective studies that investigate the role of ABA in patients with RA-ILD.

Nakashita retrospectively evaluated the effectiveness and safety of abatacept in 16 RA-ILD patients. None of them experienced a worsening of ILD after one year, while two patients showed complete resolution of the pulmonary lesions [137].

As reported above, in 2015, Curtis et al. evaluated the incidence of ILD and the risk of hospitalization in a large cohort of RA patients exposed to TNFi or other biologic drugs. They found no significant differences in the risk of ILD and its related complications between patients exposed to tocilizumab, rituximab, or abatacept compared with TNFi therapies [110].

In a Spanish, retrospective, multicenter, non-controlled study on 63 RA patients with ILD treated with ABA, two-thirds of them remained stable, while one-quarter experienced improvement of the dyspnea after a mean follow-up of 9.4 ± 3.2 months. In the meantime, FVC remained stable in almost two-thirds of patents and improved in one out of five patients assessed. Additionally, DLCO remained stable in almost two-thirds and showed improvement in a quarter of the patients assessed. At 12 months, ILD was stable at HRCT in 11/22 patients in whom a chest scan was performed, improved in eight and worsened in three [138].

Mochizuki T. showed deterioration of ILD in 8.4% of 131 RA patients treated with ABA for at least one year. On the other hand, ILD improved in 14.5% of 55 patients with ILD at baseline. Worsening of ILD was associated with the concomitant use of MTX at multivariate logistic regression analysis. [139].

Recently, Kurata et al. evaluated the association between different bDMARDs and new-onset or worsening of RA-airway disease and RA-ILD. Pre-existing airway disease was an independent risk factor for ILD exacerbation or appearance after the start of bDMARDs, namely TNFi, tocilizumab, and ABA. Moreover, ABA was an independent protective factor for RA-ILD exacerbation [131].

Finally, in 2020, we published data from a retrospective multicenter Italian study evaluating the evolution of ILD in 44 Italian RA-ILD patients treated with abatacept for at least six months. FVC and DLCO remained stable or increased in 86.1% and 91.7% of patients, respectively, while HRCT was stable or improved in 81.4% of them. Previous and concurrent treatments, serology, age, sex, joint and lung disease duration were not associated with the outcome at univariate analysis [140].

**Figure 2:**
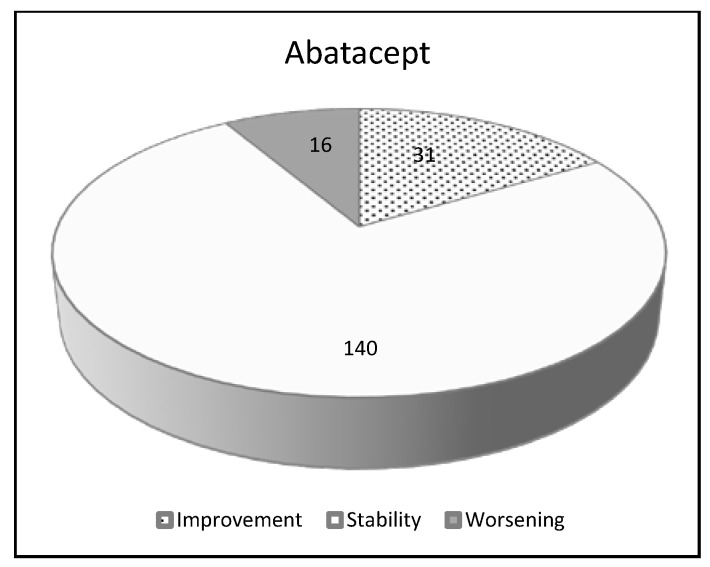
Pulmonary effects of abatacept in RA-ILD patients: a review of the literature.

Of interest, a preliminary small clinical trial is ongoing to assess the feasibility of a larger controlled study to evaluate the safety of ABA in RA-ILD (APRIL study, NCT03084419) (Table 5 and Figure 2).

### 4.3. Interleukin-6 Inhibitors

#### 4.3.1. Tocilizumab

The proinflammatory cytokine IL-6 shows profibrotic effects antagonizable by IL-6R blockade [141], suggesting a potential benefit of this therapeutic strategy in RA-associated pulmonary fibrosis. Anyway, data on its use in RA-ILD is anecdotal and conflicting.

For example, tocilizumab (TCZ) as monotherapy was found to stabilize or even improve ILD in a case-series of four RA patients, [142] and two previous case reports described similar observations [143,144].

Improvement or stabilization of lung function in 75% of cases was described also in a retrospective national multicenter study of 28 RA-ILD patients treated with TCZ with or without MTX [145].

On the other hand, adverse lung effects have been also reported after the use of TCZ. In particular, Wendling described the worsening of pre-existing ILD in a patient after 23 infusions of TCZ as monotherapy and subsequent improvement of symptoms and HRCT findings after its withdrawal [146].

Moreover, other reports correlate TCZ with AE of pre-existing interstitial lung disease, even with fatal outcome [147,148]. The retrospective case-control study by Akiyama et al. aimed to identify risk factors for AE of ILD during TCZ treatment in patients with RA. Of 78 patients, six developed AE. Univariate analysis showed that only disease activity was a risk factor for AE. [148].

Data from real-life post-marketing surveillance show a good safety profile for TCZ in a Japanese population of RA-ILD patients [149,150].

In an interim analysis, the presence of ILD was a risk factor for AE and serious infections [149]. In 2014, among 7901 patients, ILD was recorded in 38 (22 patients had ILD at baseline, while 16 developed ILD during treatment). Twenty-four of 38 patients had previously received other biologics. Of the 38 patients, 14 improved, 12 recovered and seven died. In a multivariate logistic regression analysis, the risk factors for ILD deterioration were advanced age (≥65 years) and previous or concurrent ILD at baseline [150].

**Figure 3:**
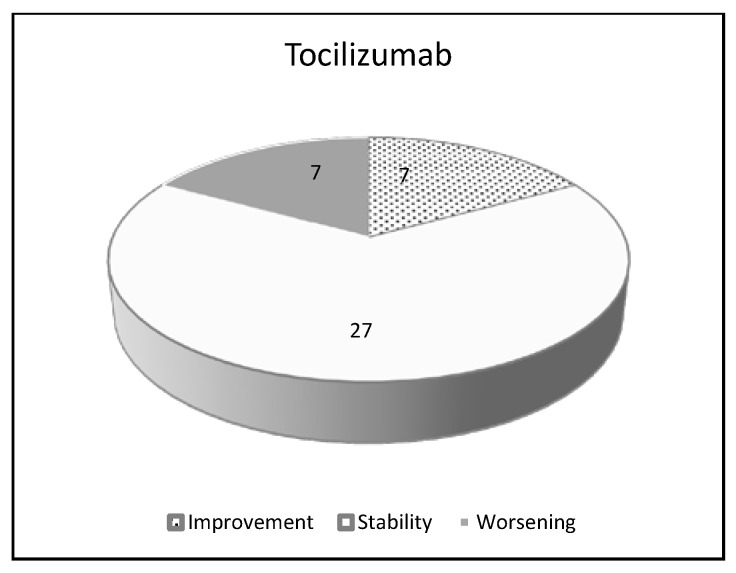
Pulmonary effects of tocilizumab in RA-ILD patients: a review of the literature.

Of interest, the incidence rate of ILD (0.5%) found in Japan for TCZ was similar to that recorded for infliximab (0.5%), adalimumab (0.6%), and etanercept (0.6%) (Table 6 and Figure 3) [98,103,124].

#### 4.3.2. Sarilumab

No new onset of ILD was described in RA patients treated with sarilumab during pre-marketing clinical trials. However, all the enrolled patients were evaluated for the presence of lung disease and a pre-existing ILD was considered as an exclusion criterion.

### 4.4. Rituximab

According to some retrospective data e anecdotal case reports [151,152,153,154], rituximab (RTX) is usually considered a safe therapy for ILD including severe refractory forms [155]. However, a meta-analysis of biological therapies in CTD noted that RTX was associated with an increase of non-infectious parenchymal lung disease [156]. Furthermore, lung toxicity is widely described for RTX in hematological patients [156,157,158,159,160].

In a prospective study of 33 RA patients, the use of RTX resulted in a DLCO decline in 22% of the patients. Even if no cases of symptomatic lung injury were observed, the progressive DLCO decline suggested the presence of subclinical RTX-induced pulmonary toxicity [161].

In 2012, Hartung et al. firstly described a 66-year-old patient with severe RA-ILD successfully treated with RTX after the failure of prednisolone and CYC [153].

In the retrospective study by Keir et al., 33 patients received RTX for ILD related to CTD, two of them with RA. The Authors concluded that RTX may be an effective therapeutic option in these cases, even if the highest proportion of patients with a categorical improvement was seen in the group with IIM. Data on RA patients cannot be deduced [162].

In 2019, Duarte et al. described 17 RA-ILD patients treated with RTX. After a 12-month follow-up, all patients with OP or NSIP (*n* = 12) demonstrated improvement or stability of PFTs and HRCT. Regarding patients with UIP pattern, 2/3 of patients had a decline in FVC and half had HRCT worsening [15].

Moreover, Chartrand described a highly variable response in the clinical status of 15 RA-ILD patients, without significant variations of FVC over time nor a corticosteroid-sparing effect [163].

A 10-year study by Yusof et al. assessed the effects of RTX in 700 RA patients, of whom 56 (8%) had a previous diagnosis of ILD and 44 had data on lung function; pulmonary involvement improved or remained stable in 68% of cases, while 18 patients (32%) showed a progression of ILD and half of them (16%) died because of progressive ILD. Factors associated with ILD progression were radiologic UIP pattern, a previous history of lung progression, and DLCO < 46% predicted before the therapy. During the follow-up period, only three patients developed incident cases of RA-ILD (incidence of 0.4%) [154].

In 2017, Druce et al. retrospectively analyzed 352 patients with RA-ILD treated with either RTX or TNFi as first-line biologic therapy, observing no differences in survival and cause of death between the two groups [152].

In 2019, Fui et al., in another retrospective study on 14 RA-ILD patients, observed a possible effect of RTX in reducing lung function deterioration after 6 and 12 months [164].

On the contrary, Matteson showed improvement of ILD only in 1/10 RA-ILD patients treated with RTX in an open-label pilot study [165]. Finally, Becerra et al. didn’t experience respiratory improvement in 19 patients with established RA-ILD over four years, and 15 patients (66%) reported respiratory infections. [166].

**Figure 4:**
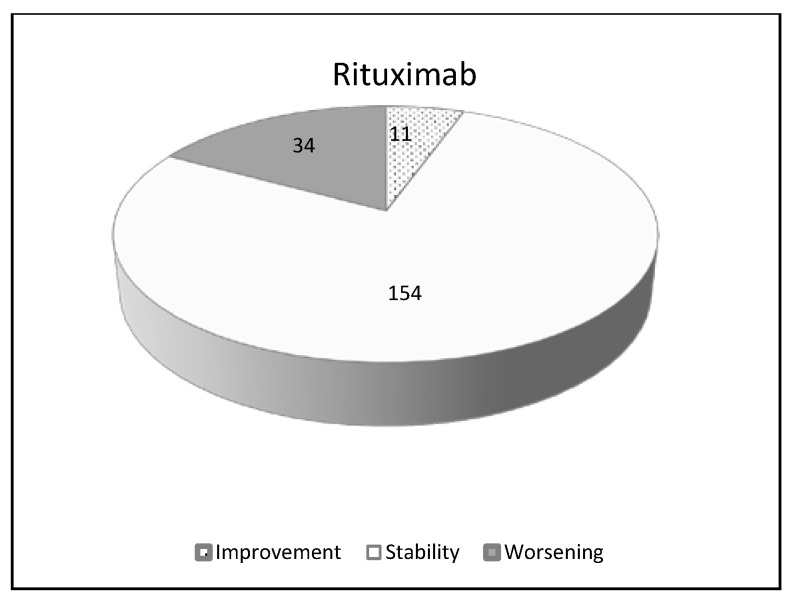
Pulmonary effects of rituximab in RA-ILD patients: a review of the literature.

A high rate of side effects was reported also by Dass in 48 patients with RA-ILD treated with RTX: three patients died, one because of pneumonia and possible acute progression of ILD. Five patients had a decline of DLCO > 10%. [167]. In another abstract, 53 patients with RA-ILD treated with RTX were analyzed. There was no substantive or significant reduction in FVC and DLCO over time; 11 patients received also CYC. Only three patients were diagnosed with new ILD after RTX. Nine patients died because of progressive ILD (Table 7 and Figure 4) [168].

## 5. Targeted Synthetic Disease-Modifying Antirheumatic Drugs (tsDMARDs)

Tofacitinib and baricitinib have been recently licensed for the treatment of RA. Tofacitinib selectively inhibits the janus-kinase (JAK) 1 and 3, while baricitinib blocks JAK1 and JAK2 pathways.

Data regarding the relationship between JAK inhibitors and RA-ILD in real life are limited [169].

In RA clinical development programs of tofacitinib and baricitinib, 0.1% of the patients developed ILD and some of them were de novo ILD [170,171].

Successful use of tofacitinib has recently been described for ILD associated with anti–melanoma differentiation–associated protein 5 (anti-MDA5)–positive amyopathic dermatomyositis [172,173].

Phase III trials of tofacitinib in combination with MTX have reported only a few cases of new-onset ILD and pulmonary sarcoidosis [174], and a combination of pulmonary fibrosis and chronic obstructive pulmonary disease have been observed in trials using tofacitinib as monotherapy [175].

A very low rate of ILD was recorded in the open-label extension of pre-marketing trials and in the post-marketing surveillance of tofacitinib: 18/2631 patients developed ILD in combination therapy with MTX and 9/1543 in monotherapy in the open-label extension of clinical trials and 15 cases on 34,223 patients/year in post-marketing surveillance, respectively [176,177]. An interim analysis of the Japanese post-marketing surveillance program of tofacitinib identified 14 cases (0.5%) with serious ILD, of which three died [178].

In three RA-ILD patients, tofacitinib was able to treat joint involvement with the stability of lung function and without pulmonary adverse events [179].

Recently, tofacitinib demonstrated its ability to facilitate the expansion of myeloid-derived suppressor cells (MDSC) and ameliorate arthritis in SKG mice, a murine model developing not only arthritis but also ILD. In SKG mice, tofacitinib significantly suppressed the progression of ILD compared to control, by increasing myeloid-derived suppressor cells and suppressing Th17 cells proliferation and differentiation [180]. On the contrary, in another in vitro study, the JAK2 inhibition, but not the selective JAK1/JAK3 pathway, significantly reduced IL-17A-induced fibrogenic response in RA-ILD patients [181].

## 6. Antifibrotic Agents

RA-ILD shares some similarities with IPF, especially in patients with a UIP pattern. It shows a similar clinical behavior, often with a progressive fibrosing phenotype, and a comparable prognosis and survival [33]. Some authors suggest that RA-ILD and IPF might also overlap in disease pathogenesis [182].

Moreover, genetic risk factors, previously well characterized in IPF, are increasingly being linked to RA-ILD. For example, the MUC5B promoter variant rs5705950, telomerase complex mutations, and short telomere lengths are also linked to an increased susceptibility to UIP pattern in RA-ILD [33,183,184].

The parallelisms between UIP in RA-ILD and IPF may suggest a plausible rationale in the use of antifibrotic therapy in these patients to treat the fibrotic process, improve outcomes and reduce lung disease progression. Moreover, given both the fibrotic and inflammatory components of this systemic disease, the combination of immunosuppressive and antifibrotic treatment can potentially be a possible future approach to this spectrum of the disease.

The INBUILD trial recently assessed the efficacy and safety of nintedanib in patients with a diagnosis of ILD other than IPF, including RA [185]. Moreover, several trials are planned or ongoing to assess the efficacy and safety of antifibrotic agents in the treatment of fibrosing ILDs other than IPF, including patients with RA (Table 8) [185,186,187,188].

### 6.1. Pirfenidone

In Europe, pirfenidone is approved for the treatment of IPF [189].

Interestingly, Pirfenidone reduces the levels of IL6 and TNF-alpha, both cytokines related to the activation of macrophages and with a proven role in RA pathogenesis [190]. Recently, it also showed an inhibitory effect on fibroblast to myofibroblast transition in RA-ILD [191].

Pirfenidone is currently under investigation in patients with RA-ILD (TRAIL1) [187]. This phase II study (NCT02808871) estimates to enroll 270 RA-ILD patients to treat with pirfenidone three times daily (2403 mg) as an add-on to existing treatment. It will evaluate a composite primary end-point with PFTs (≥10% decline in FVC or death) and other secondary outcomes (relative decline in DLCO, FVC, the incidence of AE, dyspnea scores, safety, and tolerability). No preliminary data are available yet.

### 6.2. Nintedanib

Nintedanib is approved for the treatment of IPF, it has been shown to slow down the decrease in FVC and to reduce the number of AE [189]. In vitro, nintedanib demonstrated its efficacy in reducing both pulmonary fibrosis and joint disease in female SKG mice with RA [192].

Recently, interesting results have been observed in the INBUILD study. This double-blind, placebo-controlled, phase 3 trial aimed to evaluate the efficacy and safety of nintedanib in progressive fibrosing ILD secondary to other conditions than IPF. The most frequent diagnoses were chronic hypersensitivity pneumonitis (26.1%) and autoimmune ILD (25.6%), including also RA-ILD patients. The patients who received nintedanib had a slower annual rate decline of FVC over a 52-week period in ILD than placebo. Of interest, the results were similar in patients with UIP-like fibrotic pattern or other radiological/histological patterns. However, data on specific diseases associated with ILD are not still available [185].

In 2018, a first case report described a 74-year-old man diagnosed with RA-ILD (UIP pattern) treated with nintedanib. The use of nintedanib resulted in decreased coughing together with a reduction in FVC decline, from −11.6%/year to −5.2%/year. [193].

## 7. Conservative Therapy

Conservative treatment may be advisable for patients with a mild and non-progressive disease or contraindications to pharmacological treatments, such as multiple comorbidities, advanced age or frailty syndrome. Non-pharmacological treatments usually include pulmonary rehabilitation, psychological and educational support.

### 7.1. Smoking Cessation

Cigarette smoke is implicated in the pathogenesis of RA-ILD, but also in inducing and worsening both the severity of articular disease and lung damage. Additionally, much evidence suggests that smoking reduces the drug response and survival in RA patients. In particular, some studies demonstrated a poorer response to TNFi and a reduced chance of achieving low disease activity for heavy smokers, especially for infliximab. Both smoking status and pack-year history at the start of therapy have been associated with a low response to treatment [194].

On the contrary, more recently the CIMDORA study did not find any correlation between cigarette smoking and the effectiveness of certolizumab pegol in 218 RA patients [195].

Finally, a recent metaanalysis found no high-quality studies that investigate the evidence of the effects of smoking cessation on disease activity in RA patients [196].

However, given its well-established role in the development of RA and pulmonary toxicity and its association with the severity of the diseases, smoking cessation should be strongly encouraged. The support of anti-smoking counseling centers and the possible use of nicotinic replacement therapy should be considered in all patients with RA.

### 7.2. Pulmonary Rehabilitation

The usefulness of pulmonary physical rehabilitation in RA-ILD is yet undefined. However, in idiopathic ILD, it has a short-term beneficial effect on dyspnea, functional exercise capacity, and quality of life [197,198]. However, in RA-ILD patients, pulmonary rehabilitation may be compromised by the functional joint limitations related to the underlying disease.

### 7.3. Oxygen Supplementation

Oxygen supplementation can be a major palliative therapy to improve the quality of life in patients with severe lung disease, reducing respiratory symptoms during daily activities.

No data directly address the use of long-term oxygen supplementation in patients with IPF or RA-ILD.

### 7.4. Vaccination

Corticosteroids and immunosuppressants, as well as the presence of ILD, are associated with a high risk of serious infection in RA patients [49,50].

Influenza and anti-pneumococcal vaccines should be proposed for all RA patients. Some Authors also recommend prophylaxis against pneumonia by Pneumocystis Jirovecii for all patients in immunosuppressive therapy [3].

### 7.5. Comorbidities

Specific treatment is recommended in case of comorbidities that can worsen the clinical course of the disease, for example, pulmonary hypertension, chronic obstructive pulmonary disease (COPD), gastro-oesophageal reflux, and sleep apnoea.

In particular, COPD can be associated with ILD in smokers or former smokers and it can determine the presence of mixed histological/radiological patterns (Table 2), such as “combined pulmonary fibrosis and emphysema” (CPFE). Patients with CPFE often have a low DLCO and a higher risk for pulmonary hypertension and lung malignancy [199].

In IPF and RA-ILD patients, Antoniou et al. found that emphysema was associated with lower pack-year smoking histories than in control groups and with coarser pulmonary fibrosis, suggesting a possible pathogenetic link with smoking for both diseases [200]. No data are available regarding the response to therapy in CPFE subtype of RA-ILD. Finally, COPD could increase the infective risk in patients treated with conventional or biologic DMARDs [50].

## 8. Lung Transplant

Lung transplantation may be an option in end-stage RA-ILD. However, there are few studies evaluating post-transplant outcomes in RA-ILD patients. The survival rates of 10 patients with RA-ILD who had undergone lung transplantation were similar to patients with IPF [201].

In ILD related to CTD or RA, other extrapulmonary disease manifestations may complicate or contraindicate transplant procedures.

Recently, in a Northern Spanish study, CTD-ILD patients (including RA) showed a lower frequency of acute graft rejection than IPF, but also a lower 5-year cumulative survival rate [202].

Finally, a retrospective cohort compared survival, acute and chronic rejection, and extrapulmonary organ dysfunction after transplantation in patients with non-scleroderma connective tissue-related lung disease (NS-CTLD) (including RA) and IPF. The Authors found no significance between NS-CTLD and IPF. So, in appropriately selected candidates, NS-CTLD should not be considered a contraindication to lung transplantation [203].

## 9. Acute Exacerbation of Ra-Ild

Acute exacerbation (AE) is a life-threatening condition defined as rapidly deteriorating respiratory symptoms within a 1-month period with newly developed bilateral ground-glass opacities and/or consolidations on chest CT scans, superimposed on a background pattern consistent with fibrosing ILD [204]. Other than IPF, AE can also complicate secondary forms of ILD, such as RA, CTD, and chronic hypersensitivity pneumonia [205,206,207,208,209,210,211].

In RA-ILD patients, older age at ILD diagnosis, UIP pattern, and MTX have been reported as the major risk factors for AE development [212]. AE of RA-ILD can occur at any time during the disease, and occasionally it can represent the onset manifestation of ILD [205,206,207,208,209,210,211]. It has a poor prognosis and high mortality, similar to AE in IPF. Therefore, early diagnosis and referral might be important for the patient’s prognosis.

Currently, there is no evidence-based data on effective therapies in AE-ILD. Usually, corticosteroid therapy is empirically used, with or without immunosuppressive agents and antibiotics. [205,206,207,208,209,210,211,213,214,215].

Ota et al. [214] retrospectively reviewed 12 RA-ILD patients with AE treated with corticosteroids. Tacrolimus was added in three cases, cyclosporine in four and CYC in another five patients. Pulmonary function and HRCT alterations significantly improved in all cases but the CYC group had a better life prognosis, while two patients in the cyclosporine group and one patient treated with corticosteroids alone died for a relapse of AE.

Toyoda retrospectively reviewed 10 patients with CTD-ILD and AE, including six RA-patients. All patients were treated with antimicrobial agents and high dose corticosteroids, whereas CYC or tacrolimus was added only when a poor response to corticosteroids was observed. The median survival time after onset AE was significantly longer in patients treated with corticosteroids only [209].

In 2019, we investigated the incidence of AE in a population of patients with CTD-ILD; in this context, two patients with RA and AE were enrolled and treated with a high dose of corticosteroids: one patient died while the other survived [211].

Finally, two patients with AE successfully treated with nintedanib without corticosteroids or immunosuppressants have been described [215,216].

## 10. Biomarkers and Response to Treatment

Although there are some contrasting data, many biomarkers have been investigated as possible predictors of appearance or progression of lung involvement in RA [217]. In particular, Krebs von den Lungen 6 (KL-6) could reflect the severity of ILD as assessed through HRCT and PFT parameters. Its potential role has been evaluated prevalently in Asian patients both in diagnosis and monitoring lung involvement [217,218]; a high value of KL-6 has been associated with mortality and usual interstitial pneumonia (UIP) pattern at HRCT [218]. Moreover, an increase of KL-6 has been associated with a progression of ILD over time as a possible marker of treatment response [139,219].

Many other biomarkers have been evaluated in RA-ILD, such as matrix metalloproteinase 7 (MMP-7), pulmonary and activation-regulated chemokine, and surfactant protein D (SP-D). In 113 RA patients, a combination of age, sex, smoking, rheumatoid factor, and anticyclic citrullinated peptide antibodies was strongly associated with RA-ILD; the inclusion of MMP-7, pulmonary and activation-regulated chemokine, and SP-D significantly increased the areas under the curve from 0.88 to 0.97 for the identification of patients with ILD. Interstingly, similar trends were seen for both clinically evident and subclinical RA-ILD [220].

Finally, a specificity of 90% for RA-ILD has been reported by Harlow for serum autoantibodies targeting citrullinated heat shock protein 90 alpha (Hsp90α) or beta (Hsp90β) [221].

## 11. Proposal for Patient Management and Treatment

The optimal therapeutic regimen of RA-ILD has not been determined as no large randomized controlled trials are yet available.

Treatment options for RA-ILD are further complicated by the implication of almost all drugs used for RA in pulmonary toxicity, and the lack of evidence for their efficacy in the treatment of ILD. Moreover, immunosuppressive drugs employed in CTD or antifibrotic drugs approved for IPF are not effective on the articular manifestation of the disease.

Furthermore, the substantial variability in RA-ILD clinical presentation (subclinical, progressive, slow progression, non-progressive, chronic, acute exacerbation, etc.), histopathologic subtypes, and disease course make it difficult to speculate about one milestone therapeutic approach.

Therefore, the development of guidelines for RA-ILD treatment remains an open challenge.
The treatment of RA-associated ILD should be tailored for each patient after the evaluation of:
  - age, gender, comorbidities;
  - progression and severity of the lung involvement (symptoms, PFTs, DLCO, HRCT);
  - histopathologic or HRCT pattern of ILD;
  - activity and severity of joint disease;
  - other extra-articular manifestations.

A multidisciplinary approach, including at least a rheumatologist, pulmonologist, and radiologist, is necessary to optimize therapy and follow-up strategies. A multidisciplinary evaluation has been confirmed as having a high level of confidence in particular for the diagnosis of IPF and CTD-ILD including RA-ILD [222].

Moreover, all RA patients should be considered at risk for ILD and the evaluation of lung involvement during the routine clinical assessment is mandatory. Indeed, an early diagnosis is needful to ensure that each patient receives appropriate treatment for their particular clinical phenotype and to avoid the use of drugs potentially involved in ILD worsening. In this regard, we recently proposed the use of VECTOR as a simple, non-invasive and inexpensive tool for the screening of RA patients suspected for ILD [223].

A tight follow-up must be recommended in RA-ILD patients, utilizing periodic assessment of respiratory symptoms, PFTs, DLCO and HRCT.

In patients with active joint disease and subclinical non-progressive ILD, current therapy with DMARDs (including MTX and TNFi) should be continued to achieve low disease activity; while in patients starting a new DMARD, the use of ABA, JAK inhibitors, IL6 inhibitors or RTX could be appropriate [39,40,109]. However, we caution about the use of RTX in patients at a high risk of infections of the lower respiratory tract [49,50,166]. The association with MTX should be evaluated in every single patient.

In patients with progressive lung disease and mild articular involvement, immunosuppressants, such as CYC and MMF, should be considered [51,52,53,54,55,56,61,62,63,64]. On the other hand, the recent data about nintedanib allow us to suppose the future use of antifibrotic agents in ILD secondary to RA [185].

**Figure 5:**
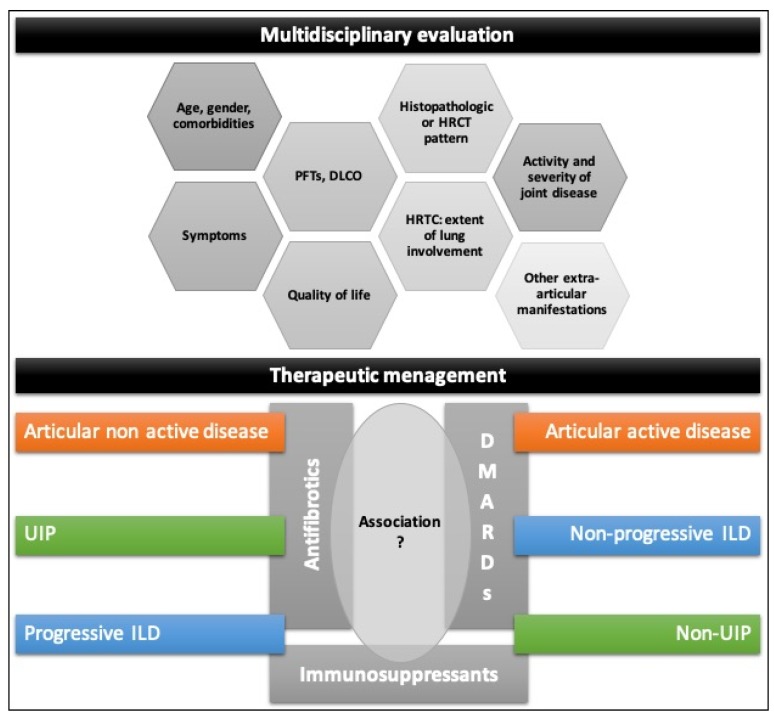
Proposed framework for the management and treatment of RA-ILD patients. Therapeutic choice in patients with RA-ILD should derive by a multidisciplinary approach including a rheumatologist, pulmonologist, and radiologist. Use of DMARDs (mainly ABA, JAK inhibitors, IL6 inhibitors or RTX) should be evaluated according to the joint disease activity, while the progression of lung involvement and, possibly ILD pattern, can influence the decision to use immunosuppressants or, in selected patients, an antifibrotic drug.

Finally, in patients with progressive ILD and active joint disease, combination therapy with antifibrotic agents and bDMARDs could represent an interesting future research field (Figure 5).

## 12. Conclusions and Research Agenda

ILD is one of the most common extra-articular manifestations of RA, and its management is challenging, for the deterioration of quality of life, the high mortality, and utilization of healthcare resources.

Unfortunately, ILD is often underrated, particularly in its early and subclinical stages, and the majority of available studies on this topic are retrospective and based on low-quality data.

The real epidemiology of ILD in RA patients is unknown, and no proteomic or serologic biomarkers are available to improve our armamentarium for both diagnostic and prognostic purposes. Non-homogeneous and sometimes discordant results regarding risk factors for RA-ILD have been described and, finally, randomized controlled clinical trials to support therapeutic decisions in RA-ILD patients are still missing.

In summary, there is an urgent need for prospective studies to clarify these crucial points in the field of RA-ILD.

In this framework, possible future directions of research include the prognostic value of sub-clinical RA-ILD; the development of screening programs to achieve early diagnosis of RA-ILD; prospective studies to discover biomarkers and predictors of lung involvement in RA.

Moreover, well-designed therapeutic trials are mandatory, and future research could move to more personalized management and treatment of RA-ILD patients, for example, to evaluate the possible concomitant use of DMARDs and anti-fibrotic agents.

Recently, antifibrotic medications are supposed to have relevance across various ILD subtypes, not only in the UIP pattern (INBUILD). Ongoing clinical trials in patients with non-IPF fibrosing ILDs [185,186,187,188,224,225,226,227] and RA-ILD patients [185,186,187,188] (Table 8) will provide valuable insights into the progression of these diseases in well-characterized populations.

Finally, the cooperation between multidisciplinary groups with different experiences may be advisable for further well-designed studies on this topic. Efforts to develop a research network comprising dedicated centers with both respiratory and rheumatology interest in RA-ILD should also be made to deliver important new knowledge of this condition.

## Figures and Tables

**Table 1:** Lung involvement in Rheumatoid Arthritis.

**Interstitial lung disease**	
UIP	
	NSIP, OP, DIP, LIP, mixed disease
**Airways disease**	
Bronchiectasis	
Bronchiolitis	
	Bronchiolitis obliterans
	Follicular bronchiolitis
	Panbronchiolitis
	Chronic small airway obstruction
	Cricoarytenoid arthritis
**Rheumatoid nodules**	
generally, in subpleural areas, single or multiple, solid or cavitary, range in size	
**Pleural disease**	
Pleuritis	
Pleural effusion	
	Pleural thickening
	Lung entrapment and trapped lung
Pneumothorax	
**Vascular disease**	
	Pulmonary hypertension
	Primary (related to underlying vasculitis)
	Secondary (associated to ILD)
Vasculitis	
	Haemorrhagic alveolitis
	Venous thromboembolism
**Caplan syndrome**	
it occurs in patients with both RA and pneumoconiosis	
**Lower respiratory tract infection**	
Common bacterial	
Opportunistic infection (pneumocystis jirovecii)	
Fungal	
Mycobacterial	
**Amyloidosis**	
**Apical fibrobullous disease**	
**Lung cancer**	
**Drug toxicity**	
Nsaids	
	Diffuse pulmonary infiltration
	Eosinophilic pneumonia
	ARDS
	Bronchospasm
	Infection/Pneumonitis
	Noncardiogenic pulmonary edema
Glucocorticoids	
	Infection/Pneumonitis
	Cyclophosphamide and
Mycophenolate mofetil	Infection/Pneumonitis
	Fibrosis
	Noncardiogenic pulmonary edema
Methotrexate	
	Hypersensitivity pneumonitis
	Infection
	New onset or exacerbation of ILD
	Noncardiogenic pulmonary edema
	Bronchospasm
Leflunomide	
	Hypersensitivity pneumonitis
	Infection
	New onset or exacerbation of ILD
	Other conventional DMARDs
	Infection/Pneumonitis
	Obliterative bronchiolitis
	New onset or exacerbation of ILD
	Drug-induced lupus
	Biologic DMARDs
	Infection/Pneumonitis
	Noncardiogenic pulmonary edema
	New onset or exacerbation of ILD
	Drug-induced lupus

**Table 2:** Histologic classification and typical features of idiopathic interstitial pneumonia,.applicable to rheumatoid arthritis RA-ILD interstitial lung disease [9,10,11,12,13,14,15,16,17,18,19].

Histologic Pattern	Prevalence in RA	Pattern of Distribution	Radiographic Findings
UIP: Usual interstitial pneumonia	8–66%	Peripheral, subpleural, basal	Reticular opacities; honeycombing; minimal ground-glass opacity; architectural distortion
NSIP: Nonspecific interstitial pneumonia	19–57%	Peripheral, basal, symmetric	Extensive ground-glass opacity; irregular linear opacities; traction bronchiectasis; subpleural preservation
RB: Respiratory bronchiolitis	0–42%	Principally upper fields, centrilobular	Bronchial wall thickening; centrilobular nodules; ground-glass opacities
Mixed forms and unclassifiable interstitial pneumonia	0–11%		Coexisting patterns of interstitial fibrosing and other lung disease, e.g., emphysema
OP: Organizing pneumonia	0–11%	Subpleural, peribronchial	Focal ground-glass opacities; consolidations; reversed halo sign
DAD: Diffuse alveolar damage	0–11%	Diffuse or focal	Consolidations; ground-glass opacities; traction bronchiectasis
DIP: Desquamative interstitial pneumonia	rare	Lower fields, predominantly peripheral	Ground-glass attenuation; cysts; reticular opacities
LIP: Lymphoid interstitial pneumonia	rare	Predominantly in the upper lung fields	Thin-walled cysts; centrilobular nodules; ground-glass attenuation; peribronchovascular septal thickening
PPFE: Idiopathic pleuroparenchymal fibroelastosis	rare	Peripheral, upper fields	Pleural thickening; subpleural fibrotic changes

**Table 3:** Pulmonary effects of immunosuppressants and conventional disease-modifying anti-rheumatic drugs (cDMARDs) in RA-ILD patients: a review of the literature.

**Cyclophosphamide**		
		Number of patients 89
Author, year (Ref)	Article type	
Chang HK, 2002 [86]	case report	1
Schupp JC, 2016 [57]	retrospective study	7
Fu Q, 2018 [60]	retrospective study	81
Other articles *		
Song JW, 2013 [46]	na	84
Zhang G, 2015 [56]	na	23 CTD-ILD
		
**Mycophenolate Mofetil**		
		Number of patients 29
Author, year (Ref)	Article type	
Saketkoo LA, 2008 [63]	case series	3
Fischer A, 2013 [62]	retrospective study	18
Oldham JM, 2016 [81]	retrospective study	8
Other articles *		
Zhang G, 2015 [56]	na	23 CTD-ILD
		
**Methotrexate**		
		Number of patients 72
Author, year (Ref)	Article type	
Rojas-Serrano J, 2012 [65]	retrospective study	18
Rojas-Serrano J, 2017 [72]	retrospective study	54
		
**Leflunomide**		
		Number of patients 12
Author, year (Ref)	Article type	
Rojas-Serrano J, 2012 [65]	retrospective study	12

**Azathioprine**		
		Number of patients 27
Author, year (Ref)	Article type	
Cohen JM, 1977 [79]	case report	1
Ishida T, 2012 [80]	case report	1
Rojas-Serrano J, 2012 [65]	retrospective study	10
Oldham JM, 2016 [81]	retrospective study	15
Other articles *		
Song JW, 2013 [46]	na	84
		
**Penicillamine**		
		Number of patients 7
Author, year (Ref)	Article type	
van der Schee AC, 1989 [85]	open trial	7

**Cyclosporine**		
		Number of patients 8
Author, year (Ref)	Article type	
Puttick MP, 1995 [88]	case report	1
Ogawa D, 2000 [87]	case report	1
Tokano Y, 2002 [89]	pilot study	4
Chang HK, 2002 [86]	case report	1
Ishida T, 2012 [80]	case report	1
Other articles *		
Song JW, 2013 [46]	na	84

**Tacrolimus**		
		Number of patients 11
Author, year (Ref)	Article type	
Yamano Y, 2018 [48]	retrospective case series	11

* Cumulative data on more diseases or drugs. Patients not included in the evaluation of lung outcome. Legend: na = not available.

**Table 4:** Pulmonary effects of TNFi in RA-ILD patients: a review of the literature.

TNF Alpha Inhibitors		
		Number of patients 96
Improvement	47	48.4%
Stability	35	36.1%
Worsening	15	15.5%
		
Author, year (Ref)	Article type	
Schultz R, 2001 [118]	case report	1
Vassallo R, 2002 [107]	case report	1
Bargagli E, 2004 [111]	case report	1
Antoniou KM, 2007 [112]	prospective case series	3
Wang Y, 2011 [119]	case report	1
Komiya K, 2011 [114]	case report	1
Nakashita T, 2014 [109]	retrospective review	46
Detorakis EE, 2017 [108]	prospective study	42
Other articles *		
Kurata I, 2019 [131]	retrospective study	30

* Cumulative data on more diseases or drugs. Patients not included for the evaluation of lung outcome.

**Table 5:** Pulmonary effects of abatacept in RA-ILD patients: a review of the literature.

	Abatacept	
		Number of patients 187
Improvement	31	16.6%
Stability	140	74.9%
Worsening	16	8.5%
		
Author, year (Ref)	Article type	
Wada T, 2012 [135]	case report	1
Mera-Varela A, 2014 [134]	case series	4
Nakashita T, 2014 [109]	retrospective review	3
Nakashita T, 2016 [137]	retrospective study	16
Ye W, 2017 [136]	case report	1
Fernández-Díaz C, 2018 [138]	retrospective study	63
Mochizuki T, 2019 [139]	retrospective study	55
Cassone G, 2020 [140]	retrospective study	44
Other articles *		
Kurata I, 2019 [131]	retrospective study	12

* Cumulative data on more diseases or drugs. Patients not included in the evaluation of lung outcome.

**Table 6:** Pulmonary effects of tocilizumab in RA-ILD patients: a review of the literature.

Tocilizumab		
		Number of patients 41
Improvement	7	17.0%
Stability	27	65.8%
Worsening	7	17.0%
		
Author, year (Ref)	Article type	
Mohr M, 2011 [144]	case report	1
Wendling D, 2013 [146]	case report	1
Nakashita T, 2014 [109]	retrospective review	9
Picchianti Diamanti A, 2017 [143]	case report	1
Manfredi A, 2018 [142]	case series	4
Manfredi A, 2019 [145]	retrospective study	28
Other articles *		
Koike T, 2014 [150]	Post-marketing data	22
Kurata I, 2019 [131]	retrospective study	7

* Cumulative data on more diseases or drugs. Patients not included in the evaluation of lung outcome.

**Table 7:** Pulmonary effects of rituximab in RA-ILD patients: a review of the literature.

Rituximab		
		Number of patients 201
Improvement	11	5.4%
Stability	154	76.6%
Worsening	34	16.9%
		
Author, year (Ref)	Article type	
Dass S, 2011 [167]	abstract	48
Matteson EL, 2012 [165]	open-label pilot study	7
Hartung W, 2012 [153]	case report	1
Kabia A, 2015 [168]	abstract	53
Chartrand S, 2016 [163]	case series	15
Yusof, 2017 [154]	retrospective observational study	44
Fui A, 2019 [164]	retrospective study	14
Duarte AC, 2019 [15]	retrospective study	17
Other articles *		
Becerra E, 2012 [166]	abstract	19
Keir GJ, 2014 [162]	retrospective study	2

* Cumulative data on more diseases or drugs. Patients not included in the evaluation of lung outcome.

**Table 8:** Clinical trials of antifibrotic agents for the treatment of fibrosing ILDs other than IPF, including patients with RA.

Trial Number (Ref)	Study Name	Phase, Design, Population	Patients	Duration	State
NCT02999178 (extension NCT03820726) [185]	Inbuild	Phase III efficacy and safety of nintedanib in patients with PF-ILD	663	52 w	Completed Extension in fieri
EudraCT 2014–000861-32 DRKS00009822 [186]	Relief	Phase II Efficacy and safety of pirfenidone as an add-on to existing treatment for progressive, non-IPF lung fibrosis	374	48 w	Completed
NCT02808871 [187]	Trail1	Phase II Efficacy and safety of pirfenidone as an add-on to existing treatment in patients with RA-ILD	270 estimated	52 w	Recruiting
NCT03843892 [188]	na	Expanded access program to provide nintedanib to patients with non-IPF ILD who have no alternative treatment possibilities	na	na	Available

Legend: na = not available.

## Abbreviations

ABA	ABATACEPT
AE	Acute exacerbation
ANTI-MDA5	Anti–melanoma differentiation–associated protein 5
AZA	Azathioprine
BDMARDS	Biologic disease modifying anti-rheumatic drugs
CDMARDS	Conventional disease modifying anti-rheumatic drugs
COPD	Chronic obstructive pulmonary disease
CTD	Connective tissue diseases
CTD-ILD	Connective tissue diseases-realated interstitial lung disease
CYC	Cyclophosphamide
DLCO	Diffusing capacity of the lungs for carbon monoxide
DMARDS	Disease modifying anti-rheumatic drugs
FVC	Forced vital capacity
HRCT	High-resolution computer tomography
ILD	Interstitial lung disease
IPF	Idiopathic pulmonary fibrosis
JAK	Janus-kinase
LEF	Leflunomide
MDA5	Melanoma differentiation–associated protein 5
MDSC	Myeloid-derived suppressor cells
MMF	Mycophenolate mofetil
MTX	Methotrexate
NICE	National Institute for Health and Care Excellence
NS-CTLD	Non-scleroderma connective tissue-related lung disease
NSIP	Nonspecific interstitial pneumonia
OP	Organizing pneumonia
PFTS	Pulmonary function tests
RA	Rheumatoid arthritis
RA-ILD	Rheumatoid arthritis-related interstitial lung disease
RTX	Rituximab
SSC-ILD	Systemic sclerosis-related interstitial lung disease
TCZ	Tocilizumab
TNFI	Tumour necrosis factor alpha inhibitor
TSDMARDS	Targeted synthetic disease modifying anti-rheumatic drugs
UIP	Usual interstitial pneumonia
